# Colonisation history of a vector species of veterinary interest: phylogeography of *Culicoides imicola* (Diptera: Ceratopogonidae) in the Indian Ocean revealing low genetic structure

**DOI:** 10.1051/parasite/2026044

**Published:** 2026-09-14

**Authors:** Lorn Ribon-Chaudat, Karine Huber, David Bru, Hemant Bhoobun, Brice Derepas, Marlène Dupraz, Khouaildi Bin Elahee, Diana P. Iyaloo, Romain Girod, Karien Labuschagne, Luciano Tantely, Thierry Baldet, Claire Garros

**Affiliations:** 1 ASTRE, CIRAD, INRAE, Université de Montpellier Montpellier France; 2 CIRAD, UMR ASTRE F-34398 Montpellier France; 3 Livestock and Veterinary Division, Ministry of Agro-Industry and Food Security Reduit Mauritius; 4 CIRAD, UMR ASTRE, Plateforme Technologique CYROI F-97491 Sainte-Clotilde Reunion Island France; 5 Vector Biology and Control Division, Ministry of Health and Wellness Curepipe Mauritius; 6 Medical Entomology Unit, Institut Pasteur de Madagascar Antananarivo 101 Madagascar; 7 ARC – Onderstepoort Veterinary Research, EPV Onderstepoort South Africa

**Keywords:** Biting midge, Orbivirus, Phylogeography, Population genetics, Indian ocean

## Abstract

*Culicoides imicola* (Diptera: Ceratopogonidae) is the main historical vector of livestock viruses such as bluetongue virus (BTV), Epizootic Haemorrhagic Disease virus (EHDV) and African Horse Sickness virus (AHSV), in the Afrotropical region. Despite its wide distribution across Africa, the Mediterranean basin and parts of Asia, the genetic structure and colonisation history of this species in the South–West Indian Ocean (SWIO) territories remain poorly understood. The aim of this study was to investigate how passive wind-driven dispersal and recent animal movements associated with human colonisation may have shaped the genetic structure of this vector. We used two mitochondrial markers (cytochrome oxidase 1 and cytochrome oxidase 2) and characterized genetic structure of populations from 4 territories. In addition, phylogenetic analyses were conducted with *COI* sequences from 11 territories bordering the Indian Ocean retrieved from GenBank database. Phylogenetic analyses on *COI* sequences suggest no clear lineage or genetic grouping among the 11 territories. In the SWIO, concatenated *COI* and *COII* gene analyses show significant genetic differentiation between the four territories, especially between Mauritius and Reunion where populations exhibit signs of demographic expansion. The haplotype network suggests multiple events or potential colonisation pathways, supporting the hypothesis that animal trade is the primary means of introduction from the continent or Madagascar to Mauritius and Reunion. This work serves as a basis for understanding the risk of introduction of viruses transmitted by *Culicoides* into the island territories of the SWIO and highlights the need to strengthen practices in animal trade in the region.

## Abstract (fr)

*Culicoides imicola* (Diptera : Ceratopogonidae) est le principal vecteur historique de virus affectant le bétail, tels que le virus de la fièvre catarrhale ovine (FCO), le virus de la maladie hémorragique épizootique (MHE) et le virus de la peste équine (PE), dans la région afrotropicale. Malgré sa vaste répartition en Afrique, dans le bassin méditerranéen et dans certaines parties de l'Asie, la structure génétique et l'histoire de la colonisation de cette espèce dans les territoires du sud-ouest de l'océan Indien (SOOI) restent mal connues. L'objectif de cette étude est d'examiner comment la dispersion passive par le vent et les mouvements récents d'animaux liés à la colonisation humaine ont pu façonner la structure génétique de ce vecteur. Nous avons utilisé deux marqueurs mitochondriaux (cytochrome oxydase 1 et cytochrome oxydase 2) et caractérisé la structure génétique de populations provenant de quatre territoires. De plus, des analyses phylogénétiques ont été réalisées à partir de séquences du gène *COI* issues de 11 territoires bordant l'océan Indien, obtenues via la base de données GenBank. Les analyses phylogénétiques des séquences *COI* ne révèlent aucune lignée ni aucun regroupement génétique distinct entre ces 11 territoires. Dans la zone SOOI, les analyses basées sur les séquences concaténées des gènes *COI* et *COII* montrent une différenciation génétique significative entre les quatre territoires, en particulier entre l'île Maurice et La Réunion, où les populations présentent des signes d'expansion démographique. Le réseau d'haplotypes suggère l'existence d'événements multiples ou de voies de colonisation potentielles, étayant l'hypothèse selon laquelle le commerce d'animaux constitue le principal vecteur d'introduction depuis le continent ou Madagascar vers l'île Maurice et La Réunion. Ces travaux constituent une base pour comprendre le risque d'introduction de virus transmis par les *Culicoides* dans les territoires insulaires du SOOI et soulignent la nécessité de renforcer les pratiques liées au commerce d'animaux dans la région.

## Introduction

Biting midges of the genus *Culicoides* (Diptera: Ceratopogonidae) are small haematophagous insects that play a key role in transmitting several arboviruses that infect livestock and humans on a global scale [60]. To date, studies on viruses transmitted by *Culicoides* species in the South-West Indian Ocean (SWIO) islands remain limited [1, 13, 17, 12, 74, 46]. In this region, the main veterinary issues related to orbiviruses are caused in particular by the bluetongue virus (BTV) and epizootic haemorrhagic disease virus (EHDV), both of which affect cattle, sheep, goats and deer. These two diseases present a challenge to the ruminant sector as they cause recurrent outbreaks with clinical cases (in both imported breeds and local rustic herds) [12]. Furthermore, epizootic haemorrhagic disease (EHD) also poses an economic threat to farming and breeding of the Rusa deer (*Cervus timorensis rusa*) in Mauritius and Reunion [46]. Deer populations in both Mauritius and Reunion were introduced from Indonesia in the seventeenth century for their meat [31]. While the local epidemiological situation of these diseases has been described in Mauritius (including Rodrigues) and Reunion using serological and virological data [12, 13, 46, 74], the situation in other territories in the region remains largely unknown. In Madagascar, only BTV serotype 2 has been identified [1], although additional serotypes may be circulating undetected. Five BTV serotypes (BTV-4, BTV-9, BTV-11, BTV-15, and BTV-19) and one EHDV serotype (EHDV-6) have been identified in Mayotte [17].

Recently, African horse sickness virus (AHSV, which is also *Culicoides*-borne) has demonstrated an ability to extend beyond its established geographical distribution and poses a threat to new regions. AHSV affects equids and has high mortality rates in horse populations. It is endemic to southern Africa [11], but has recently shown a high dispersal potential through host mobility, as shown by outbreaks in Thailand and Malaysia in 2020 [48]. This was already described in the 1980–1990s when the AHSV emerged in Spain, Portugal, and Morocco following the importation of zebras from Namibia [59, 72]. The scarce knowledge of the epidemiological status in an area with high animal and human mobility highlights the potential risk introduction of new serotypes of BTV, EHDV, or AHSV being introduced to the SWIO territories.

*Culicoides imicola* Kieffer, a species belonging to the *Culicoides* genus, is a historically competent vector of BTV, EHDV, and AHSV in the Afrotropical and Mediterranean regions [61]. It is found throughout continental Africa, from southern Africa [57] to the Mediterranean border [9, 34, 64], as well as in Central and West Africa [53]. The oriental distribution front of *C. imicola* is uncertain due to high species diversity and morphological identification difficulties [7, 58]. It has also been reported in India [36], Laos [39], Thailand [45], Vietnam [86], and southern China [19]. Beyond these points, including the Australasian region, *C. imicola* is not reported.

In the SWIO islands, the species has been reported to be present in at least four territories (Madagascar, Reunion, Mauritius, and Mayotte) [3, 16, 29, 42, 46], but its presence is not reported in the Seychelles. In Reunion, *C. imicola* has been shown to be abundant and dominant at low altitudes [16, 32] while in Mauritius, it is the most abundant and widespread *Culicoides* species [42]. In Mayotte, the species has only been reported at a limited number of sites, and with low abundance [29]. No faunistic inventories of the *Culicoides* genus have been published for the other territories.

Regardless of whether they split from Gondwana around 150 million years ago (Madagascar) or emerged as volcanoes during the last 10 million years (Mauritius, Comoros and Reunion), the islands of the SWIO did not host ruminants (cattle, sheep, goats and deer) before the first human settlement. The first cattle were introduced to Madagascar in the 1st–5th centuries and to the Mascarene Islands (Mauritius, Rodrigues, and Reunion) in the 17th century [2, 8]. A genomic study suggests that Malagasy cattle populations originated from an admixture event involving multiple populations of admixed African and Indian Zebu (*Bos indicus*) around the 12th century [55]. Then, during the 17th and 18th centuries, Malagasy zebus were introduced to Reunion and Mauritius, where they were later crossbred with *Bos taurus* of European origin. Furthermore, the Rusa deer, which are native to Indonesia, were introduced to Mauritius and Reunion in the 17th century [31]. This information highlights the diversity of sources from which ruminants were introduced to the SWIO area. While these historical or contemporary livestock trade movements were sourced from the oriental African coast [72] and could explain the introduction of BTV, EHDV viruses, and their associated vector species, the hypothesis of potential introduction via the long-range passive dispersal of infected *Culicoides* cannot be discounted. Importantly, the long-distance dispersal of *Culicoides* over waterbodies by wind has been documented between regions such as Northern Europe, the Mediterranean basin, and Australia [10, 20, 22, 25]. This phenomenon could also apply to the SWIO island territories, which are geographically close.

Despite the widespread distribution of *C. imicola*, only a few studies have examined the genetic structure of the species using mitochondrial and nuclear markers [43, 65]. Both studies revealed that African populations along the Indian Ocean coast share the same genetic cluster based on *COI* sequences [43]. However, the population genetic structure of *C. imicola* has never been investigated in the Oriental part of its distribution range. Furthermore, local and regional diversity in the SWIO islands has only been studied to a limited extent, with few individuals and missing territories.

This study aimed to explore the genetic structure of *C. imicola* in the Indian Ocean region (from the Indian Ocean coast to Southeast Asia), paying particular attention to the SWIO island territories, which were colonised relatively recently by human and ruminant populations. We hypothesise that the species’ genetic structure may have been shaped by both passive dispersion through winds and dispersion through cattle related to human migration. To explore this, we genotyped 345 *C. imicola* individuals collected from four territories (Madagascar, Reunion, Mauritius, and South Africa), using the mitochondrial cytochrome oxidase subunit 1 (*COI*) and cytochrome oxidase subunit 2 (*COII*) genes. We also included published sequences of *C. imicola* from the Oriental region (11 countries), resulting in a complete dataset of 522 sequences from 11 territories.

## Material and methods

### Ethics

Field collections were approved by the local authorities in each country.

### Sampling

Between 2019 and 2024, insects were sampled at 15 sites across four territories in the south–west Indian Ocean and southern Africa (Reunion, Mauritius, Madagascar, and South Africa) over a restricted period of no more than two weeks using black light suction traps (Onderstepoort Veterinary Institute design), which were set up near livestock or horses (Table 1) for 24 h. The trapping protocol was described in Grimaud *et al*. (2019) [32] and subsampling was conducted as with a modified protocol described by Van Ark & Meiswinkel (1992) [84]. After the collection, adult midges were preserved in 70% ethanol. *Culicoides imicola* individuals were identified and sexed under a binocular microscope using published reference descriptions of the species [15]. Both male and female insects were used for sequencing.

**Table 1:** Geographical location of the *C. imicola* sampling sites, with the number of sequences analysed for each data set. = sequences from a different genetic cluster [42].

Locality (+ site code)	Territory	Sampling year	Latitude	Longitude	SWIO dataset (*COI* + *COII*)	**Greater Indian Ocean dataset (*COI*)**	**Territory code**	**References**
Les Avirons (RE_A)	Reunion	2023	−21.24	55.33	37	5	RE	This study
Saint-Paul (RE_I)	Reunion	2023	−21.06	55.27	31		RE	This study
Salazie (RE_J)	Reunion	2023	−21.02	55.54	31	5	RE	This study
Saint-Joseph (RE_D)	Reunion	2023	−21.38	55.61	13		RE	This study
Sainte-Anne (RE_K)	Reunion	2023	−21.09	55.74	24		RE	This study
Roche-Terre (MU_A)	Mauritius	2023	−20.02	57.65	28		MU	This study
Pamplemousses (MU_C)	Mauritius	2023	−20.11	57.58	30	5	MU	This study
Pointe des Lascars (MU_E)	Mauritius	2023	−20.09	57.70	29		MU	This study
Nouvelle Decouverte (MU_K)	Mauritius	2023	−20.38	57.56	22		MU	This study
Case Noyale (MU_R)	Mauritius	2023	−20.41	57.37	11	5	MU	This study
Ankify (MG_M)	Madagascar	2019	−22.39	46.10	15	5	MG	This study
Mahaboboka (MG_S)	Madagascar	2019	−22.90	44.34	19	5	MG	This study
Ambatry (MG_U)	Madagascar	2019	−23.85	44.41	9		MG	This study
Ambalavao	Madagascar	2015	–21.83	46.93		19	MG	[65]
Beanka	Madagascar	2010	–18.02	44.48		3	MG	[56]
Ambohimanatrika	Madagascar	2012	–18.5	47.40		7	MG	[43]
De Aar (ZA_A)	South Africa	2024	–30.65	24.01	18	5	ZA	This study
Onderstepoort (ZA_D)	South Africa	2024	–25.65	28.19	28	5	ZA	This study
Lapalala Wilderness	South Africa	2014	–23.88	28.31		11	ZA	[76]
Marekele	South Africa	2014	–24.42	27.60		5	ZA	[76]
Port Elizabeth	South Africa	2013	−33.9	25.50		8	ZA	[43]
Maputo	Mozambique	2013	–25.96	32.56		2	MZ	[4]
Maputo	Mozambique	2013	–25.96	32.56		8	MZ	[43]
Gonarezhou	Zimbabwe	2013	−21.9	31.60		8	ZW	[43]
Marigat	Kenya	2015	0.47	35.99		17	KE	[65]
Nanyuki	Kenya	2013	0.10	37.10		7	KE	[43]
Afar	Ethiopia	2004	11.57	41.27		4	ET	[56]
Oromyia	Ethiopia	2004	8.85	40.72		6	ET	[56]
Delajebo	Ethiopia	2004	8.80	40.70		6	ET	[42]
Dubai	United Arab Emirates	2005	25.20	55.30		3	UAE	[63]
Kalapatti	India	2013	11.06	77.05		5	IN	[35]
Bangalore	India	2013	13.04	77.59		1	IN	[35]
Kantharawichai	Thailand	2019	16.15	103.15		7	TH	[44]
–	Senegal	2012	12.6	–12.2		2	SN	[42]
–	Mali	2010	11	–6.6		1	ML	[42]
–	Burkina Faso	2004	11.2	−4.3		2	BF	[42]
–	Benin	2009	11.9	3.4		3	BJ	[42]
–	France	2008	43.2	6.4		2	FR	[42]
–	Spain	2012	39.5	3.1		1	ES	[42]
–	Portugal	2010	39.9	−7.4		1	PT	[42]
–	Greece	2013	41	24.7		1	GR	[42]
–	Italy	2012	39.1	16.9		1	IT	[42]
–	Morocco	2004	34.4	−6.4		2	MA	[42]
–	Italy–Sicily	2012	38	12.6		1	SI	[42]

**Figure 1:**
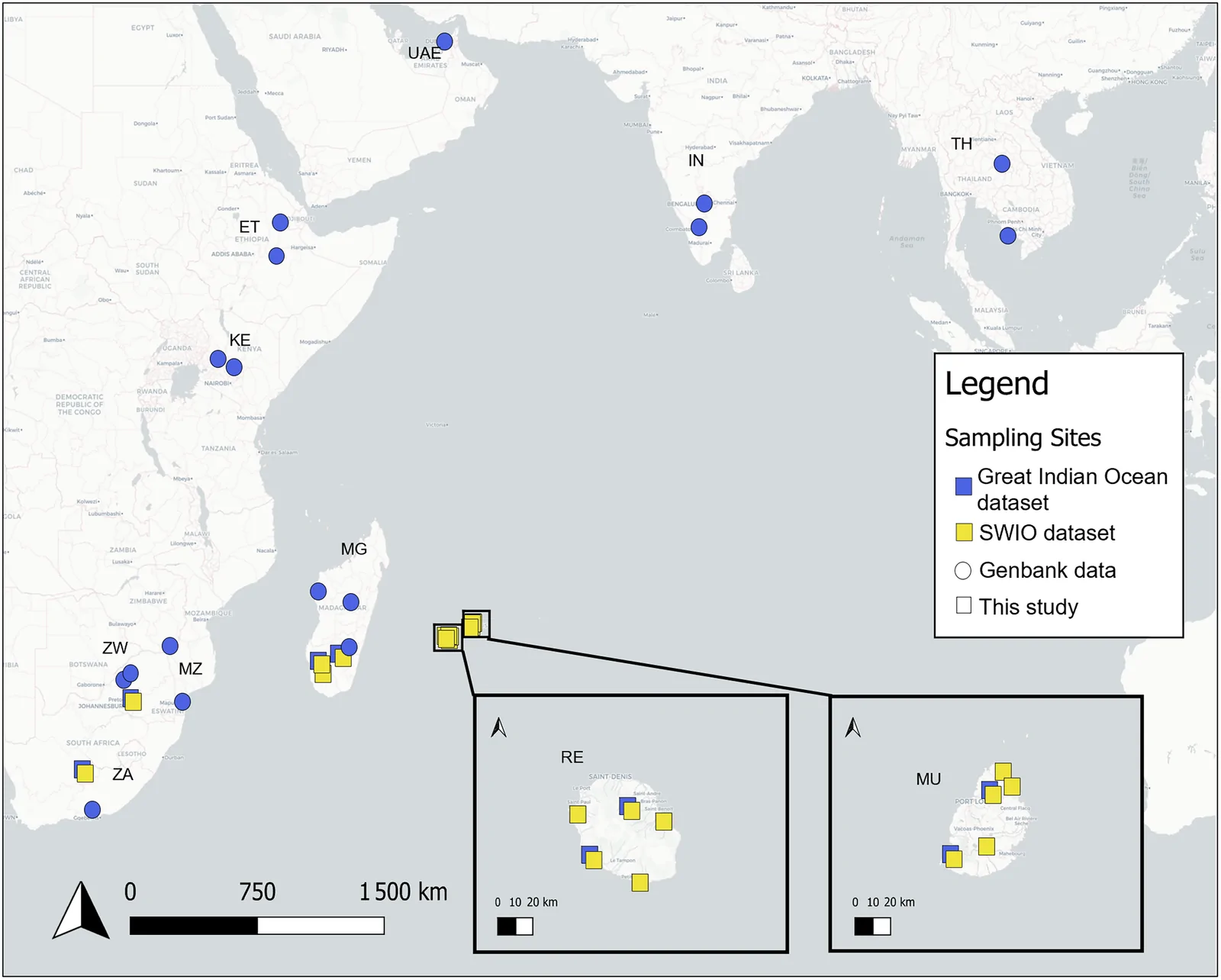
Sampling sites of *Culicoides imicola*, with both mitochondrial sequences (*COI* and *COII*) produced in this article or retrieved from the literature. Outgroup populations were not included. Country codes are described in Table 1.

In addition to the entomological surveys conducted previously, published *COI* sequences for *C. imicola* from Madagascar, South Africa, Zimbabwe, Mozambique, Kenya, Ethiopia, the United Arab Emirates, India, and Thailand were retrieved from the GenBank database and added to our dataset (Table 1, Fig. 1). Two datasets were created. The first comprises only individuals from the entomological surveys, which include Reunion, Mauritius, Madagascar, and South Africa (SWIO dataset). The second dataset includes some individuals from SWIO and the published *COI* sequences along the Indian Ocean coast (Greater Indian Ocean dataset).

### PCR amplification and sequence analyses

Genomic DNA was extracted from single midges using a NucleoSpin96 DNA RapidLyse Kit (Macherey-Nagel, Düren, Germany), according to the manufacturer’s instructions. For each individual, two mitochondrial genes, namely cytochrome oxidase subunit I (*COI*, ~648 bp) and cytochrome oxidase subunit II (*COII*, ~523 bp), were sequenced using the primers LCO1490 (5′–GGTCAACAAATCATAAAGATATTGG–3′) and HCO2198 (5′–TAAACTTCAGGGTGACCAAAAAATCA–3′) [26], COIIF20 (5′–ATGGCAACTTGAGGAMATAT–3′) and COIIR612 (5′–CGCAGATTTCTGAACATTG–3′) [56], respectively. The PCR amplification reactions for both genes were performed in a 30 μL total reaction volume containing 1× of DreamTaq Green DNA Polymerase mastermix (Thermo Scientific, Waltham, MA, USA), 2.5 mM each dNTP, 0.5 μM of each primer, and 2 μL of genomic DNA. PCR amplification conditions were as follows: an initial denaturation step at 95 °C for 3 min, followed by 5 cycles at 95 °C for 30 s; 45 °C for 30 s; 72 °C for 1 min, then 35 cycles at 95 °C for 30 s; 51 °C (for *COI*) or 54 °C (for *COII*) for 30 s; 72 °C for 1 min; and a final extension step at 72 °C for 7 min. The generated amplicons were then purified and sequenced (only in the forward direction) at the Genewiz laboratory, Azenta Lifescience (Leipzig, Germany) using the SANGER method. The obtained sequences were analysed using Geneious v.9.1.8 (Biomatters, http://www.geneious.com). Sequences were translated to verify the absence of internal stop codons and to screen for potential pseudogenes using the NCBI Open Reading Frame Finder. Samples showing low-quality chromatograms were excluded from the analyses. Sequences were aligned with the Clustal W algorithm [82] available in the software Geneious v.9.1.8 (Biomatters, http://www.geneious.com). DNA sequences for *C. imicola* were deposited in GenBank under accession numbers PZ049918–PZ050262 for *COI* and PZ067048–PZ067392 for *COII* (Supplementary material 1).

Two sequence alignments were created. The first alignment used the concatenated *COI* and *COII* genes to cover *C. imicola* populations from the SWIO (the SWIO dataset, 1,171 bp), including 15 populations from four territories. The second alignment was created using only the *COI* gene, together with published sequences, to cover a broader area of the Indian Ocean, from South Africa to Thailand (the Greater Indian Ocean dataset, 433 bp). This represents 26 populations in 11 territories. For the second alignment, sequences that were too short or uncertain were removed. Additionally, only up to ten *COI* sequences per territory from the first alignment were included in the second.

### Phylogenetic analyses

For the *COI* alignment (Greater Indian Ocean dataset), the appropriate model was selected using SMS (Smart Model Selection) in PhyML 3.0 [52]. The best-fitting model was the HKY+I model [37]. A Maximum Likelihood (ML) analysis was done with PhyML 3.0 online (available here: http://www.atgc-montpellier.fr/phyml/) with the fast likelihood-based method [33]. The tree was drawn to scale using FigTree v.1.4.4 software (http://tree.bio.ed.ac.uk/software/figtree/). Branch lengths are measured in number of substitutions per site. More sequences were selected from *C. imicola* individuals from France (FR), Spain (ES), Portugal (PT), Italy (IT), Sicily (SI), Greece (GR), Benin (BJ), Senegal (SN), Mali (ML), Burkina Faso (BF), and Morocco (MA), published by Jacquet *et al.* 2015 [43] (Table 1). These individuals were considered to belong to different genetic groups than those from the Indian Ocean region [43].

### Genetic diversity and genealogical relationships

Using the SWIO dataset (*COI* and *COII* concatenated genes), genetic diversity within and between populations and geographical areas was assessed with DnaSP 6.12.03 [54], based on the following statistics: the number of haplotypes (h), haplotypic diversity (Hd), nucleotide diversity (π), and the average number of nucleotide differences (k). We constructed a haplotype network to infer genealogical relationships among populations using the median-joining network method [6] implemented in NETWORK 4.6.1.2 software (www.fluxus-engineering.com).

### Demographic history

The genetic signature of past demographic changes was studied using neutrality tests based on Tajima’s D, Fu & Li’s D, and Fu & Li’s F with DnaSP 6.12.03. These tests were also performed on each gene. Significant negative values (i.e. a significant rejection of the null hypothesis) are expected in populations that have experienced an increase in effective population size [28, 69, 79]. We also computed a mismatch distribution test with Arlequin v.3.0 [24]. The mismatch distribution is expected to have a smooth unimodal curve in populations that have undergone rapid demographic expansion [73]. In Reunion and Mauritius, a Bayesian skyline plot implemented in BEAST v.1.8 [18] was also used to date the changes in effective population size. The analysis was conducted using the HKY substitution model, under a random local molecular clock, and a mutation rate ranging uniformly from 0.0075 to 0.0211 substitutions/site/lineage/My. A total of 10 000 000 iterations were run, a burn-in of 10%, while the parameters of the model were stored every 2 000 iterations. The posterior distributions and the graph were plotted using TRACER v1.6 software.

### Genetic structure and differentiation

Genetic structure on the SWIO dataset was assessed using a Bayesian clustering method implemented in the RhierBAPS v1.1.3 package [83] on R, derived from the hierBAPS algorithm [14]. The analysis was performed using 100 replicate runs, with a maximum number of populations (K) set to 15, corresponding to the number of currently sampled sites.

To estimate the genetic differentiation between the populations in the SWIO region, we performed an analysis of molecular variance (AMOVA) in Arlequin v3.0 [24]. In these analyses, *F*_CT_ corresponds to the component of genetic variation among groups, *F*_SC_ to the component of genetic variation among populations within groups, and *F*_ST_ to the component of genetic variation within populations. The groups were defined to represent the four territories in the SWIO (Reunion, Mauritius, Madagascar, and South Africa). In addition, we constructed a population tree based on mean uncorrected *p*-distance between populations obtained using MEGA v 11.0.13 [80]. *P*-distances and Pairwise *F*_ST_ [40] between the four territories were also calculated with MEGA 11 software and DnaSP v6.12.03, respectively. We also calculated the inter-population *F*_ST_ between the 15 populations using DnaSP v6.12.03 to determine whether geographic distance between locations may have contributed to an underlying population structure (isolation by distance). To investigate this, the *F*_ST_/(1 − *F*_ST_) values against geographic distances (km, estimated from GPS data) were plotted and the *R*^2^ coefficient calculated with R v4.4.0 software (R Core Team, 2024).

## Results

### Genetic structure in the Greater Indian Ocean

**Figure 2:**
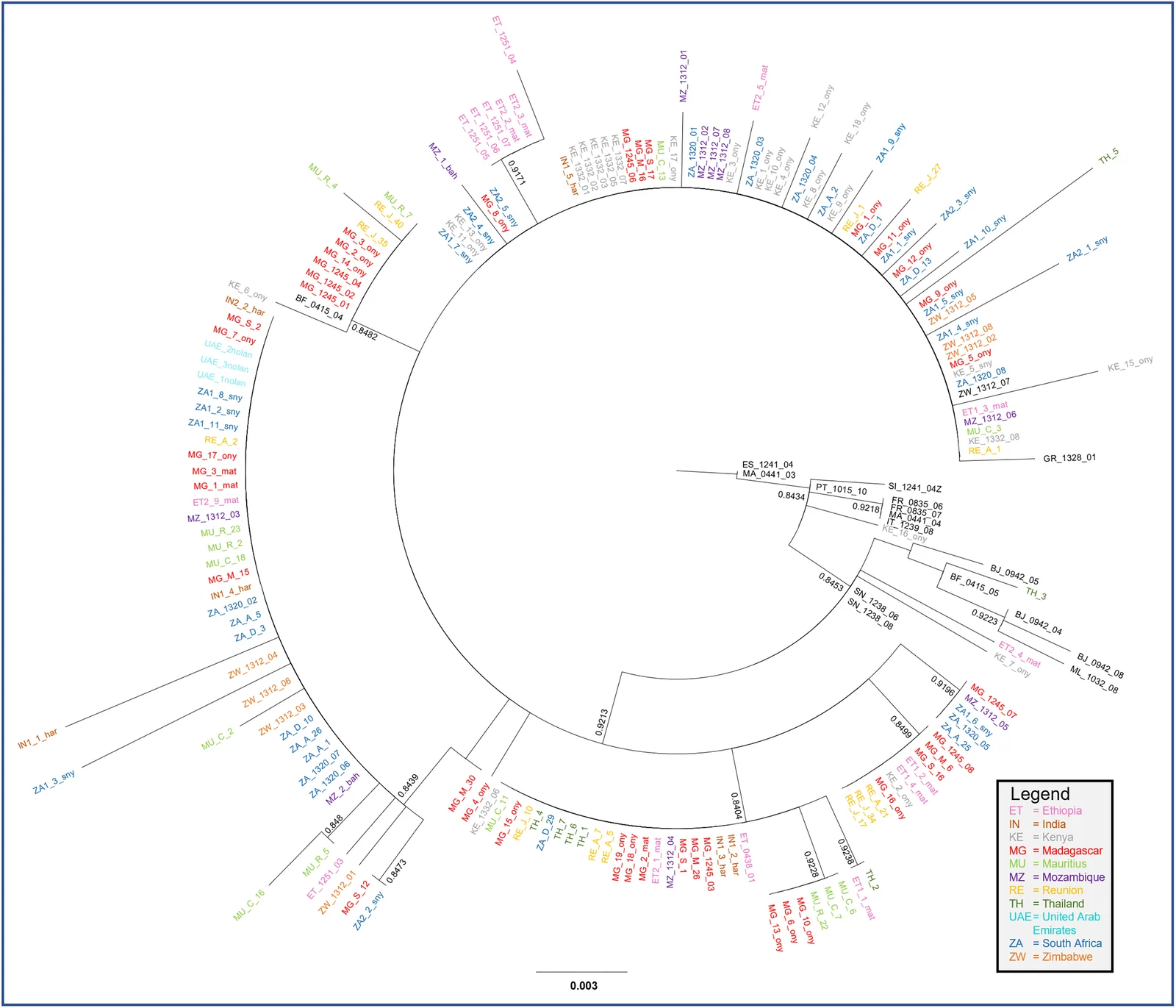
Maximum-likelihood tree of *Culicoides imicola* using *COI* sequences from the Greater Indian Ocean dataset. Numbers on branches are aLRT SH-like support values (Guindon *et al*., 2010). The individuals in black are the outgroups from other regions of the *C. imicola* range (Jacquet *et al*., 2015).

The Greater Indian Ocean alignment comprises 184 sequences (177 and seven outgroup sequences). Maximum-likelihood analysis does not reveal any clear clusters, indicating that there are no lineages or genetic groups among the 11 territories (see Fig. 2). Sequences from Oriental and Afrotropical populations are not segregated into different clusters. The ML tree shows no evidence of a relationship between any genetic clusters and geographic territories, nor any indication of the potential origin of the Mauritius and Reunion populations.

### Genetic structure in the SWIO region

**Figure 3:**
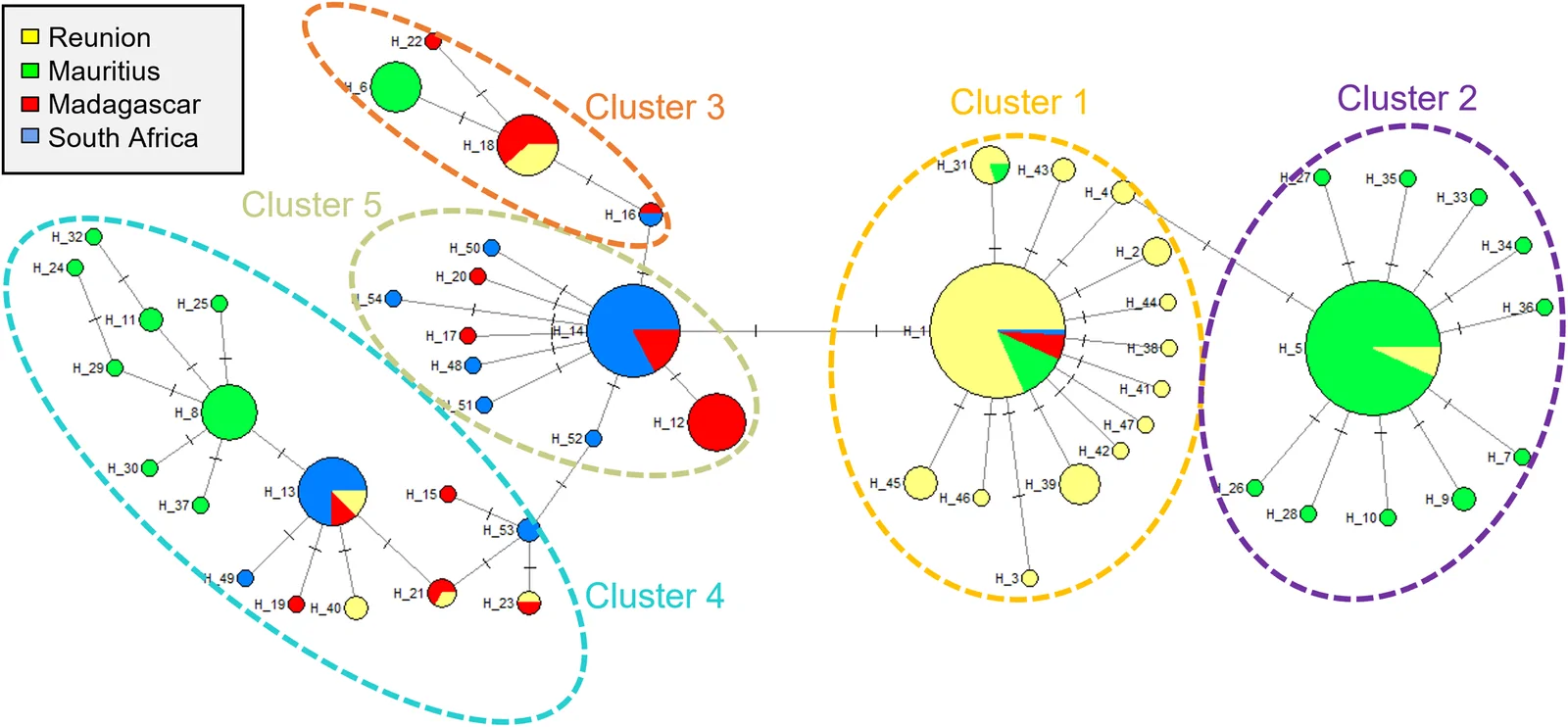
Median joining network of haplotypes for the concatenated genes *COI* and *COII* and genetic clustering from HierBAPS results for *Culicoides imicola* in the South West Indian Ocean. For each haplotype, the size of each circle is proportional to the number of sequences belonging to the haplotype. The hatch marks between circles are the number of mutations between closely related sequences. The colours represent the geographical origin of the sequences.

A total of 54 haplotypes were found in the concatenated alignment of 345 *COI* and *COII* sequences (1,171 bp) (SWIO dataset) (Fig. 3).

**Fig. S1:**
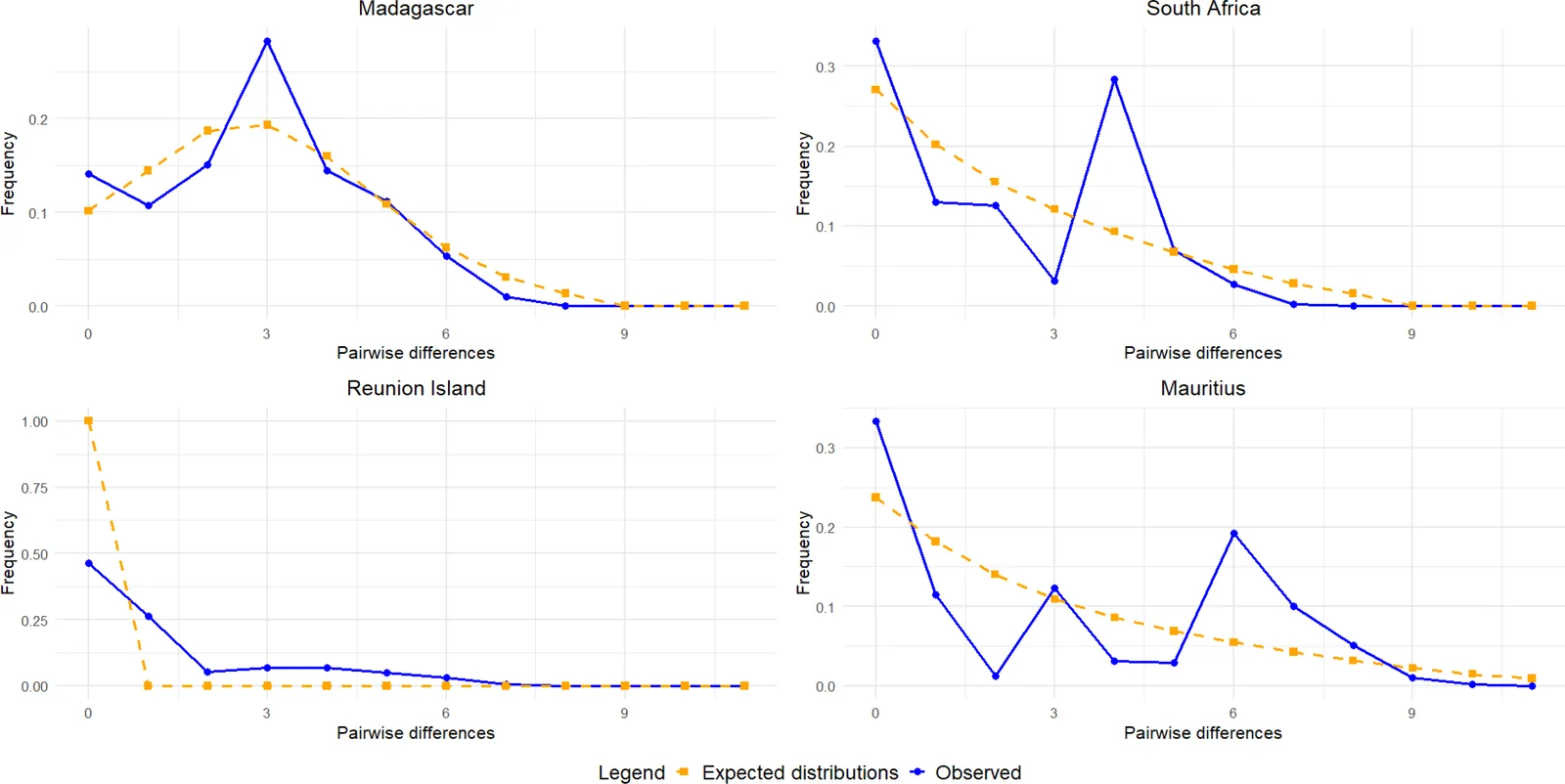
Mismatch distribution among pairwise differences among haplotypes based on the SWIO dataset (*COI* and *COII* concatenated genes) in different territories. Mismatch analyses were conducted according to a sudden expansion model with Arlequin v3.0.

**Fig. S2:**
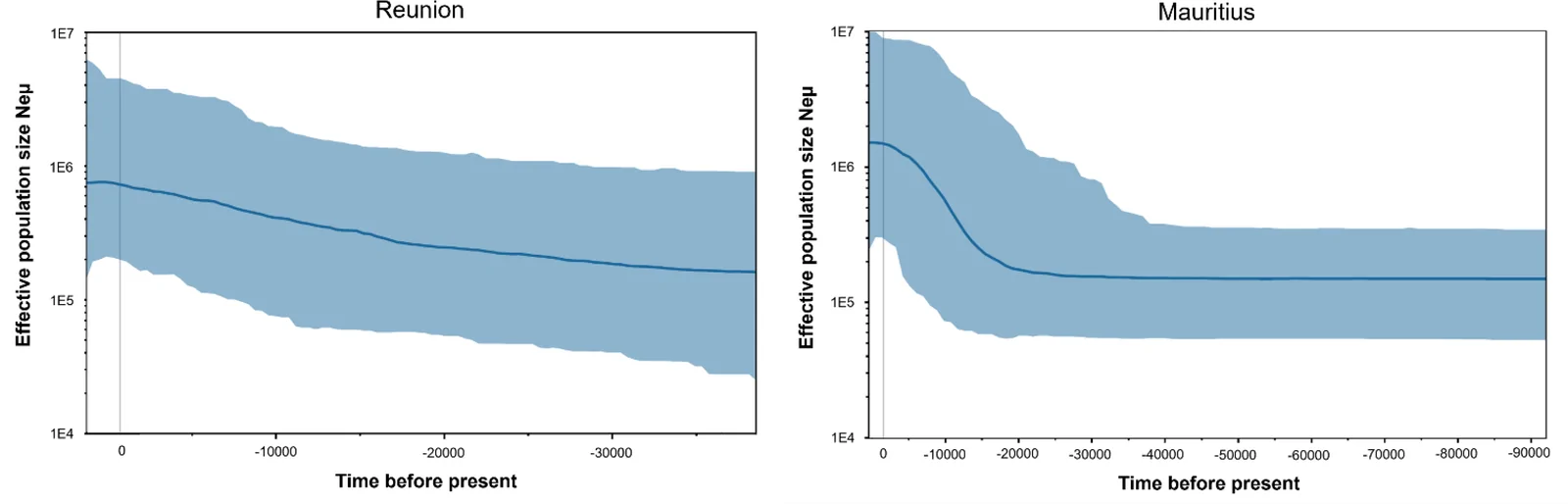
Bayesian skyline plot (BSP) based on the *COI* and *COII* combined dataset (SWIO dataset) in *C. imicola* in Reunion and Mauritius. BSPs were performed with a mutation rate of 0.0075–0.0115 substitutions/site/Myr and a random local molecular clock. The x-axis indicates times in years before present, and the y-axis shows the effective population size. The black line represents the median population size and the grey outline indicates 95% posterior intervals for the population size change.

*COII* gene alignment displayed lower polymorphism than *COI* (Supplementary material 2, Table S1). The genetic diversity indices for the *COI* and *COII* concatenated genes within territories are shown in Table 2. Haplotype diversity in South Africa (0.669) and Mauritius (0.666) was found to be lower than in Madagascar (0.860) and higher than in Reunion (0.538). Nucleotide diversity ranges from 0.0011 (Reunion) to 0.0027 (Mauritius). The neutrality tests were close to zero and non-significant, except for Reunion (Tajima’s *D* = −1.8481, *p* < 0.05) and South Africa (Fu & Li’s *D* = −2.9842, Fu & Li’s *F* = −2.6861; *p* < 0.05). The Ramos-Onsins & Rozas *R*_2_ and the raggedness *r* values were non-significant for all SWIO territories. Likewise, the mismatch distributions were not clearly unimodal for all territories, except for Reunion (Supplementary material 2, Fig. S1). The Bayesian skyline plot (BSP) suggests past demographic expansion for both Reunion and Mauritius (Supplementary material 2, Fig. S2).

**Table 2:** Descriptive statistics of genetic diversity and neutrality tests using the SWIO dataset.

Territory	*N*	Nb hap	Hd	pi	*K*	Tajima’s *D*		Fu & Li’s *D*		Fu & Li’s *F*	
						*COI*	*COII*	*COI*	*COII*	*COI*	*COII*
All	345	54	0.837	0.0028	3.260	−1.2879	−1.3283	−5.3594	−2.3987	−4.4252	−2.2837
South Africa	46	11	0.669	0.0018	2.133	−0.9227	−0.5278	−2.5826	−2.2043	−2.4130	−1.9668
Madagascar	43	13	0.860	0.0023	2.779	0.3226	0.1451	0.3507	0.1719	0,4003	0.1912
Reunion	136	20	0.538	0.0011	1.311	−1.7432	−1.4672	−2.8985	0.3951	−2.9553	−0.3004
Mauritius	120	22	0.666	0.0027	3.1563	−0.8645	−0.6345	−1.3886	−2.2590	−1.4278	−2.004

*N* = sample size (number of collected individuals); Nb hap = Number of haplotypes; Hd = Haplotype diversity; pi = nucleotide diversity; *K* = Average number of nucleotide differences; *p-value < 0.05. **p-value < 0.02.

The haplotype network suggests a relatively strong relationship between South Africa and Madagascar (Fig. 3). The pattern of genetic variation in Reunion is characterized by one dominant haplotype (haplotype H_1) and numerous rare haplotypes. This yields a signature characteristic of populations that have undergone demographic expansion. The same result was observed for Mauritius, where the dominant haplotype is H_5, but many haplotypes are distant from this haplogroup (in Clusters 3 and 4; for example, there are 10 SNP differences between H_5 and H_8).

**Figure 4:**
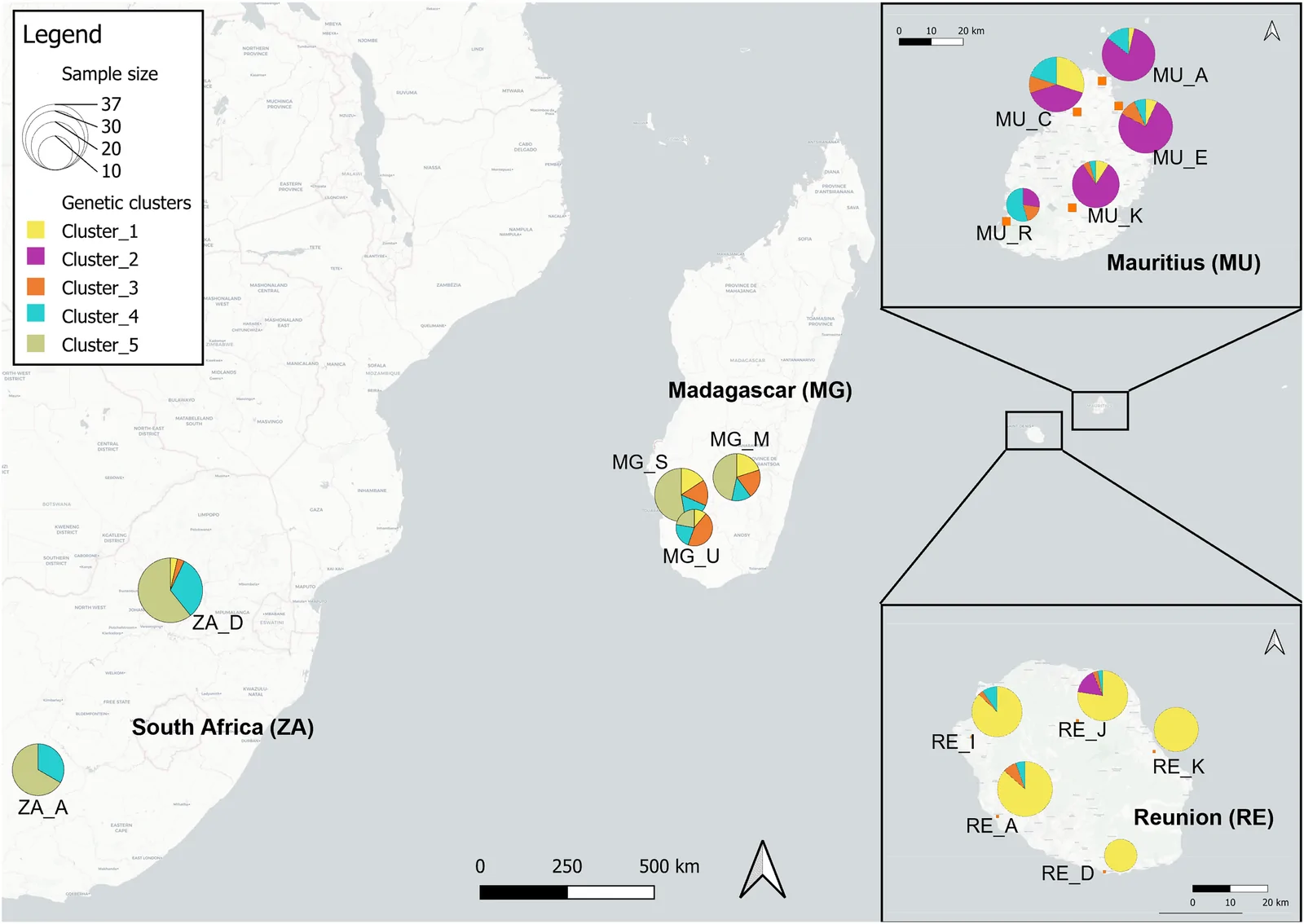
Geographic distribution of *Culicoides imicola* concatenated genes *COI* and *COII* haplotypes found in the SWIO. Pie sizes correspond to sample size for each population included in the analyses. Different colours correspond to different haplotype clusters.

Genetic clustering using hierBAPS gave an optimal number of 5 clusters (Figs. 3 and 4). Clusters 5 is prevalent in South Africa and Madagascar with 63% and 44% of individuals assigned, respectively. Reunion and Mauritius individuals belong to one different dominant cluster each: Cluster 1 for Reunion (88.2%) and Cluster 2 for Mauritius (65%). The abundances of all clusters are more heterogeneous between populations of Mauritius (especially Pamplemousses and Case Noyale populations).

The genetic distance values between the four territories using *P*-distances and *F*_ST_ methods are shown in Table 3. *P*-distance values range from 0.0023 (South Africa and Madagascar) to 0.0037 (Mauritius and Madagascar). Pairwise *F*_ST_ values vary widely between 0.1130 (South Africa and Madagascar) and 0.5468 (South Africa and Reunion), which suggests genetic proximity between South Africa and Madagascar. We can note that the *F*_ST_ value between Mauritius and Reunion populations is fairly high (0.4230).

**Table 3:** *P*-distance (below diagonal) and pairwise *F*_ST_ s (above the diagonal) between territories using COI and COII concatenated data of C. imicola in the South West Indian Ocean. Significant values are highlighted in bold.

	Reunion	Mauritius	Madagascar	South Africa
Reunion	–	**0.4230**	**0.4257**	**0.5468**
Mauritius	0.0032	–	**0.3241**	**0.3184**
Madagascar	0.0028	0.0037	–	**0.1130**
South Africa	0.0030	0.0034	0.0023	–

**Figure 5:**
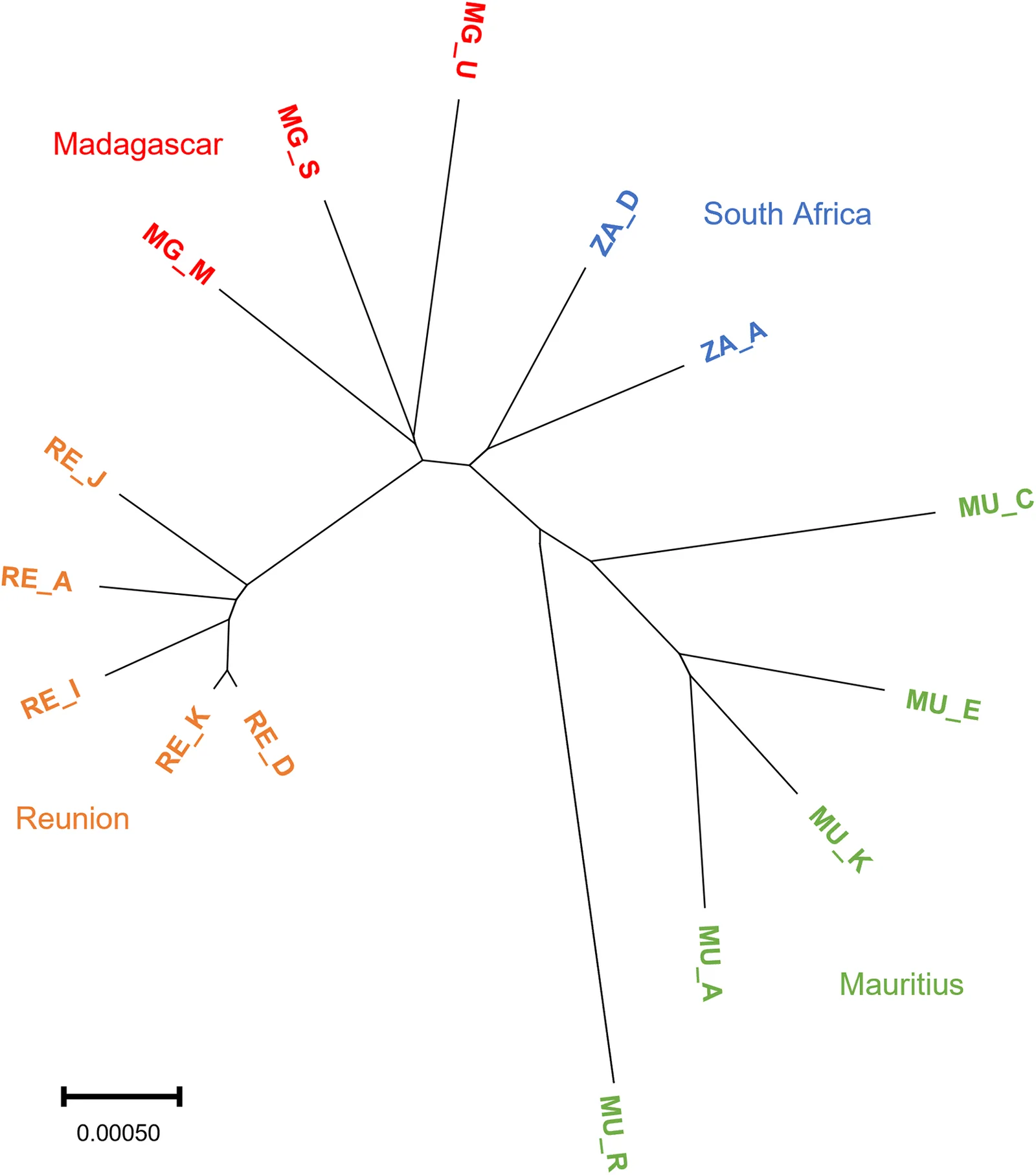
Population tree constructed with the neighbour-joining method based on the average p-distances between populations of *Culicoides imicola* using the SWIO *COI* and *COII* concatenated dataset in the South West Indian Ocean.

The AMOVA tests (Table 4) indicated relatively high intra-population variations of 42.2% (*p*-value = 0). Variation between territories was also high with 38.5% (*p*-value = 0), which suggests genetic differentiation between territories. In contrast, the proportion of variance explained within territories was low (around 6%, *p*-value = 0), indicating a certain uniformity inside a territory. In addition, we did not detect any isolation-by-distance effect between populations of the four territories based on the concatenated *COI* and *COII* markers, with a non-significant correlation between geographic distances and *F*_ST_/(1-*F*_ST_) values (*R*^2^ = 0.0289). The population tree based on pairwise genetic distances (Fig. 5) illustrates the relationships between the geographic populations, depicting generally short distances between all populations. This tree shows that the populations from Reunion and Mauritius are not closely related. Whereas populations within a territory are genetically close, Mauritius populations tends to be more distant between them.

**Table 4:** Analysis of molecular variance (AMOVA) using COI and COII concatenated data for the four territories of *C. imicola* in the SWIO. The significance tests are made with 1023 permutations.

	df	var	%	Fixation Indices	Significance tests
Between Territories	3	0.653	38.5	F_CT_ = 0.385	*p*-value = 0.000
Within Territories	11	0.028	3.8	F_SC_ = 0.061	*p*-value = 0.000
Within Population	330	0.167	57.8	F_ST_ = 0.422	*p*-value = 0.000

df = degree of freedom; var = variance components; % = percentage of variation.

## Discussion

This study aimed to explore the phylogeographic patterns of *C. imicola* in the SWIO territories using two mitochondrial markers. This species is known to disperse over large distances across waterbodies in the Mediterranean basin [44]. We sampled *C. imicola* individuals in the SWIO region and used sequences from the literature to cover the Oriental distribution area. Our findings revealed the following:

(1) Across the Greater Indian Ocean, no genetic structure was observed between African, SWIO, and oriental populations using the *COI* gene.
(2) In the SWIO region, genetic structuring clearly separated mainland African and Malagasy populations from those of Reunion and Mauritius.
(3) Significant genetic differentiation separated Reunion and Mauritius, while a subset of Mauritian haplotypes clustered more closely with African and Madagascar lineages.

Taken together, these results are consistent with a complex colonisation history of the Indian Ocean islands, involving multiple, temporally distinct colonisation events from different geographic origins (Madagascar, mainland Africa, or the Oriental range of the species). *COI* data reveal limited genetic structure at the scale of the Indian Ocean basin, likely reflecting both the relatively short length of the marker and incomplete geographic sampling, which may reduce the power to detect finer-scale differentiation and potentially introduce biases in the inferred patterns of genetic structure [62]. On another hand, genetic differentiation detected within the SWIO using concatenated *COI*–*COII* sequences – particularly between mainland Africa and Madagascar *versus* Reunion-Mauritius, and between those islands – suggests a combination of historical introductions and more recent dispersal processes. In Reunion and Mauritius, the genetic patterns observed are also consistent with recent demographic expansion following the more recent colonisation of these two islands from the 17th century onwards, potentially coinciding with the introduction of livestock and the development of farming activities, which likely increased host availability and facilitated population growth of *Culicoides*.

Historically, *C. imicola* is an Afrotropical species, widespread throughout the African continent, the Mediterranean basin and the Middle East, and also recorded in the Indian subcontinent and Far East, i.e. India [21], Southern China [88], Thailand [67], and Vietnam [86]. At the scale of the Greater Indian Ocean, *COI*-based analyses yielded results consistent with those reported by Jacquet *et al*. (2015) [43], who grouped sequences from southern Africa, Madagascar, and Reunion with those from East Africa and the United Arab Emirates. Our results further show that sequences from India and Thailand also belong to this same cluster. No evidence of cryptic diversity was detected for these different populations. Although, to our knowledge, this is the first time that oriental sequences have been compared with Afrotropical sequences, only a few sequences from the Oriental region have been obtained. These do not provide a sufficient overview of genetic diversity and structure to enable comparison and draw conclusions about intraspecific diversity.

Madagascar separated from Africa during the fragmentation of Gondwana (ca. 170–88 Ma), and haematophagous biting midges of the genus *Culicoides* diverged during the mid-Cretaceous [78]. Although some elements of Malagasy fauna are shared with Africa, the low genetic differentiation observed between East African and Malagasy *Culicoides* populations is unlikely to reflect ancient vicariant processes. Instead, this pattern, together with the high genetic diversity observed within East African and Malagasy *Culicoides* populations, likely reflects human-mediated introductions and subsequent regular exchanges between southern Africa and Madagascar. The high genetic variability in Madagascar suggests either large, demographically stable populations or multiple successful colonisation events. Indeed, the first cattle were introduced to Madagascar from East Africa and Comoros between the 1st and 5th centuries AD [2], providing new mammalian hosts that could have facilitated *Culicoides* dispersal and establishment.

The genetic patterns observed in Reunion and Mauritius differ markedly. Although the two islands are genetically differentiated from each other, their populations are also distinct from African and Malagasy populations.

On Reunion Island, populations are structured around a dominant haplotype surrounded by several low-frequency haplotypes, forming a characteristic star-like haplotype network. This type of pattern is consistent with a scenario of recent introduction followed by a demographic bottleneck and subsequent population expansion. This interpretation is further supported by a significant Tajima’s D value for Reunion and by Bayesian Skyline Plot (BSP) analyses, which suggest past demographic expansion on this island.

In Mauritius, the population is composed of individuals carrying haplotypes assigned to four clusters. Cluster 2 appears to be almost exclusively specific to Mauritius and exhibits a star-shaped distribution, suggesting local demographic expansion. Cluster 1 mainly comprises haplotypes from Reunion, whereas Clusters 3 and 4 predominantly include haplotypes from southern Africa and Madagascar. These results give rise to several hypotheses:

Firstly, Mauritius may have experienced multiple colonisation events by *C. imicola* from different source regions and at different time periods. One pathway likely originated from the African mainland and/or Madagascar, while another may have involved Reunion. The African mainland and/or Malagasy pathway is supported by Clusters 3 and 4, which include haplotypes shared across all SWIO territories, as well as a few haplotypes originating from Reunion (e.g. H13 and H18). In contrast, the pathway involving Reunion is supported by Cluster 2, which is largely specific to Mauritius and exhibits a star-shaped distribution, as well as by its genetic link with Cluster 1, which predominantly comprises haplotypes from Reunion. In addition, a migration route from more distant regions bordering the Indian Ocean cannot be excluded. Onyango *et al*. (2015) [65] reported gene flow between *C. imicola* populations from Kenya and the SWIO islands. Phylogenetic analyses of *C. imicola* along the African coast of the Indian Ocean have been used to assess this hypothesis; however, this issue could not be fully addressed due to the limited fragment length and the small sample sizes. Similar scenarios involving multiple dispersal pathways have been proposed for other *Culicoides* species, such as *C. brevitarsis*, which is thought to have colonised Australia via incursions from South Asia [81]. Comparable genetic patterns have also been described in other taxa. For instance, populations of the bumblebee *Bombus terrestris* in Madeira were found to be genetically closer to European continental populations than to those from the Canary Islands [85], highlighting how complex colonisation histories can shape insular genetic structure.

We acknowledge that additional source regions not included in the present study may also have contributed to the observed patterns. Several areas of Madagascar and other islands in the SWIO, such as Rodrigues and the Comoros archipelago (including Mayotte), could not be sampled for this study and may have provided further insights into population sources and dispersal pathways. Passive dispersal by wind and human-mediated transport have likely contributed to the colonisation of *C. imicola* in the SWIO. While wind-assisted dispersal of *Culicoides* across water bodies is well documented [10, 23, 44], past movements of human and livestock may also have facilitated dispersal, notably through the inadvertent transport of immature stages in contaminated litter or manure.

The golden orb-weaving spider (*Nephila*), a passive aerial disperser in the SWIO, provides a clear illustration of wind-mediated colonisation [51]. In the case of *Nephila*, colonisation of the Mascarene Islands (Mauritius, Rodrigues, and Reunion) appears to have originated from Madagascar: the Mascarene populations share a common “ancestral” *COI* haplotype that is closely related to, but either absent or not yet sampled, in Madagascar. Notably, the haplotype network pattern observed in *Nephila* is very similar to that observed in *C. imicola*, supporting the idea that wind-assisted dispersal could play a comparable role in shaping genetic structure across these oceanic islands.

Human-linked dispersal is less well documented, although a limited number of studies have suggested that *Culicoides* may be transported at either immature or adult stages through commercial activities [47, 63, 71, 77], or via contaminated manure and live animal movements. Such processes would be expected to result in recurrent colonisation events, leading to relatively high genetic diversity in Mauritius and the presence of haplotypes shared with putative source regions, such as southern Africa and Madagascar. Given the genetic patterns observed in our study, both wind-assisted and human-mediated colonisation pathways cannot be ruled out.

The genetic signal observed in Reunion and Mauritius suggests relatively recent demographic expansion. One possible explanation for this result could be the recent human colonisation of these island in the 17th century, together with the introduction of farm animals (cattle, goats, and sheep) or later, wild hosts like the *Rusa* deer [31]. The subsequent increase in hosts density and suitable habitats (including anthropogenic substrates linked to livestock farming [27]) could have led to the expansion of *Culicoides* population. The continuous increase in the curve for *C. imicola* populations of Reunion and the increase around 15 000 years ago for the Mauritius population, as revealed by the Bayesian Skyline Plot, suggest population growth. However, the date of expansion does not appear to correlate with human colonisation in the 17th century. One possible reason for this discrepancy is that the Bayesian Skyline Plot analysis of the mitochondrial marker does not have the temporal resolution necessary to detect demographic expansions occurring on short historical timescales, despite signals of recent expansion being visible in the haplotype network. In Mauritius, it is likely that this signal could have been exacerbated by heterogeneous spatial grouping of multiple different haplotypes [38].

Despite the short geographic distance between Reunion and Mauritius (approximately 230 km), there is low genetic similarity between populations from the two islands, with individuals from Mauritius being closer to those from South Africa. In this region, the trade winds typically blow from the southeast to the northwest, with occasional gusts from the northeast to the southwest [87], which could facilitate rare dispersal events from Mauritius to Reunion. However, our results suggest that such events are extremely infrequent, as previously observed in the Mediterranean basin [44]. Besides this, the genetic differentiation between the two islands, added to the low genetic diversity in Reunion, could result from other processes such as genetic drift and a founder effect. *F*_ST_ estimates based on Hudson’s approach should also be interpreted with caution, as they can be influenced by low levels of genetic diversity and sampling design, which may affect the precision of inferred differentiation. Overall, the observed patterns are consistent with limited gene flow between islands, although alternative explanations cannot be excluded.

Within each island, genetic homogeneity is evident, suggesting ongoing gene flow likely driven by active biological dispersal by flying *Culicoides*, which has been demonstrated up to 2.5 km in Europe [49, 50, 75]. Interestingly, the northern (MU_C) and southwestern (MU_R) populations in Mauritius are genetically more distinct from each other. Mitochondrial DNA markers have provided us with our first insights into the history of *C. imicola* in the SWIO. However, in order to better understand the colonisation process in this region, nuclear markers are needed. Mitochondrial DNA is maternally inherited, lacks recombination in animals, and has a higher mutation rate than nuclear DNA, which can lead to an incomplete or potentially biased understanding of population history and gene flow [5]. One consequence is that mitochondrial markers can be influenced by demographic processes such as genetic drift, or selective sweep [35]. Moreover, inferences based on mtDNA can be confounded by the linkage disequilibrium (co-inheritance) between heritable bacteria (endosymbionts) and the host mtDNA [41]. The bacteria *Candidatus* Cardinium hertigii (Bacteroidetes) is a maternally-inherited endosymbiont that is found in *C. imicola* in South Africa and that causes linkage disequilibrium between the endosymbiont and mitochondria [68], and therefore genetic differentiation between infected and non-infected *C. imicola* populations. One hypothesis is that if the endosymbiont is present in the SWIO, it may also play a role in shaping the genetic structure of populations there.

Our study provides some insights into the potential barrier to gene flow for *C. imicola* in the SWIO region and indicates a low risk of introduction of new BTV or EHDV serotypes by wind-borne infected *Culicoides* to and between islands. However, as Jacquet *et al.* (2016) [44] have demonstrated, even this rare event should not be overlooked. Therefore, temporal investigations may be conducted in relation to the trade wind season or extreme weather events such as cyclones. Moreover, other *Culicoides* species are involved in the transmission of BTV and EHDV in the region, as has been shown for *C. bolitinos*, *C. enderleini*, *C. kibatiensis*, or *C. grahamii* for example in South Africa [66] and in Reunion [30, 32]. These species are found in all SWIO territories [3, 4, 16, 42, 70] and may exhibit different periods of activity (as shown in Grimaud *et al.*, 2019 [33]) or different dispersal capacities, which could increase the risk of BTV, EHDV, or even AHS introduction by wind-borne infected *Culicoides*. Further analysis of the different species using mtDNA or other more discriminatory markers and meteorological modelling are required in order to better assess this risk.

## Figures and Tables

**Fig. S3:**
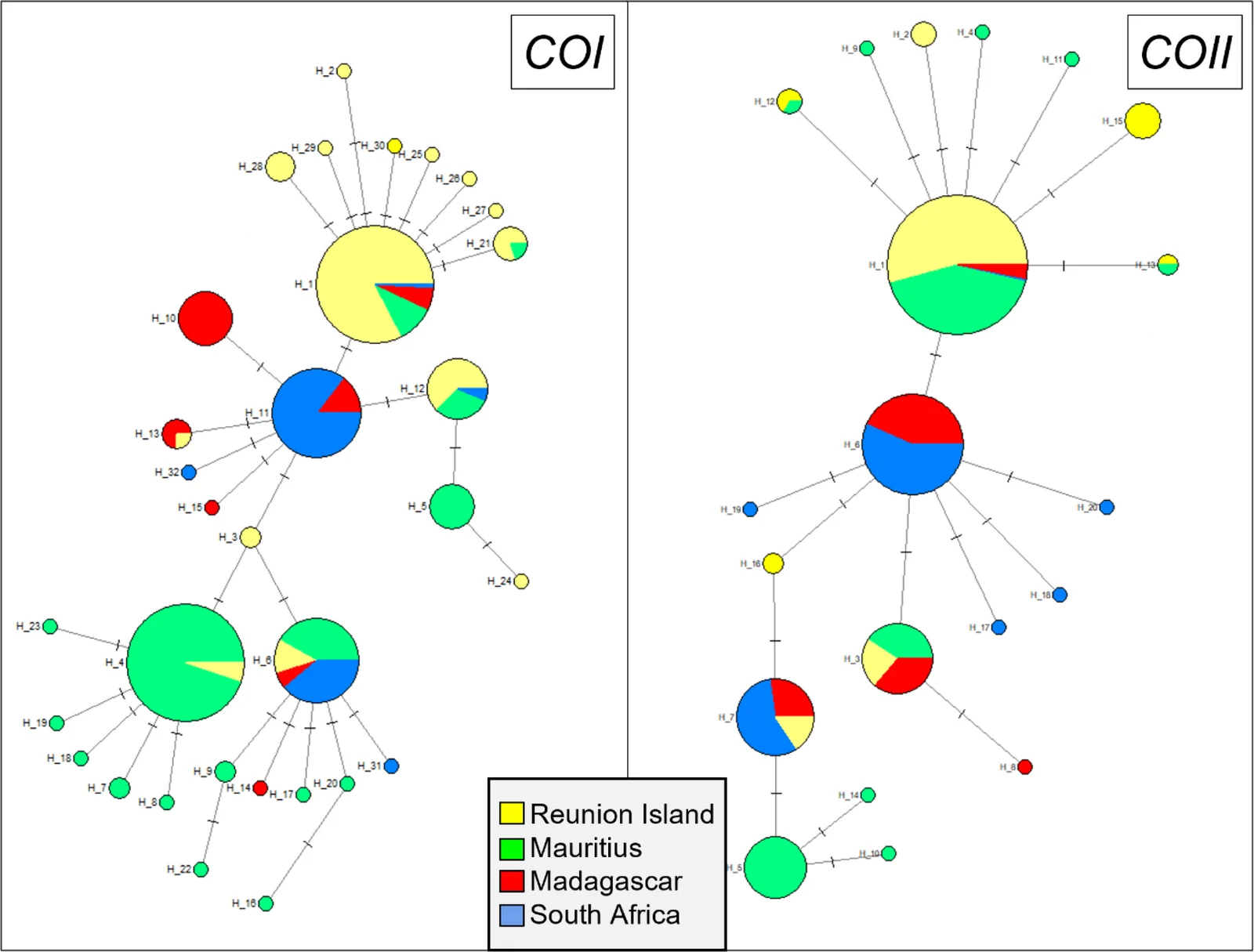
Median joining networks of haplotypes for the *COI* and *COII* genes of *C. imicola* in the South West Indian Ocean. The size of each circle corresponds to the number of haplotypes. The hatch marks between circles are the number of mutations between closely related sequences. The colours represent the geographical origin of the sequences.

## Supplementary material

*Table S1*: Description of the samples and sequence number assignment. *Table S2*: Descriptive summary of genetic diversity and neutrality tests using *COI*, *COII*, and both concatenated genes from the SWIO dataset for *C. imicola* in the South West Indian Ocean. N = sample size (number of collected individuals); Nb hap = Number of haplotypes; Hd = Haplotype diversity; pi = nucleotide diversity; K = Average number of nucleotide differences; * = *p*-value <0.05. ** = *p*-value <0.02. *Table S3*: P-distance (below diagonal) and Pairwise FSTs (above the diagonal) between populations using the *COI* and *COII* concatenated dataset (SWIO dataset) of *C. imicola* in the South-West Indian Ocean. Significant values are highlighted in bold.

The supplementary material of this article is available at https://www.parasite-journal.org/10.1051/parasite/2026044/olm.
