# Supplementary material for: Colonisation history of a vector species of veterinary interest: phylogeography of Culicoides imicola (Diptera: Ceratopogonidae) in the Indian Ocean revealing low genetic structure
Source: Parasite. 2026 Sep 14;33:50. doi: 10.1051/parasite/2026044 (PMC13574315; doi:10.1051/parasite/2026044)
Supplement: Supplementary file 1 [file parasite-33-50-s2.pdf]

Table S2: Descriptive summary of genetic diversity and neutrality tests using *COI*, *COII*, and both concatenated genes from the SWIO dataset for *C. imicola* in the South West Indian Ocean. N = sample size (number of collected individuals); Nb hap = Number of haplotypes; Hd = Haplotype diversity; pi = nucleotide diversity; K = Average number of nucleotide differences; * = *p*-value < 0.05. ** = *p*-value < 0.02

	**Territory**	**N**	**Nb hap**	**Hd**	**pi**	**K**	**Tajima's D**	**Fu & Li's D**	**Fu & Li's F**
COI & COII	**All**	345	54	0.837	0.0028	3.260	-1.3283	-5.2664**	-4.2450**
	**South Africa**	46	11	0.669	0.0018	2.133	-0.8480	-2.9842*	-2.6861*
	**Madagascar**	43	13	0.860	0.0023	2.779	0.2793	0.3336	0.3720
	**Reunion**	136	20	0.538	0.0011	1.311	-1.8481*	-1.9073	-2.2583
	**Mauritius**	120	22	0.666	0.0027	3.1563	-0.8565	-2.2414	-2.0470
COI	**All**	345	32	0.794	0.0031	1.987	-1.2879	-5.3594**	-4.4252**
	**South Africa**	46	7	0.544	0.0016	1.046	-0.9227	-2.5826*	-2.4130*
	**Madagascar**	43	8	0.817	0.0024	1.557	0.3226	0.3507	0.4003
	**Reunion**	136	14	0.401	0.0012	0.775	-1.7432*	-2.8985*	-2.9553*
	**Mauritius**	120	15	0.615	0.0027	1.766	-0.8645	-1.3886	-1.4278
COII	**All**	345	20	0.608	0.0024	1.273	-1.3283	-2.3987*	-2.2837
	**South Africa**	46	7	0.585	0.0020	2.133	-0.5278	-2.2043	-1.9668
	**Madagascar**	43	5	0.712	0.0023	1.223	0.1451	0.1719	0.1912
	**Reunion**	136	8	0.307	0.0010	0.536	-1.4672	0.3951	-0.3004
	**Mauritius**	120	10	0.452	0.0026	1.390	-0.6345	-2.2590	-2.004
