# Supplementary material for: Colonisation history of a vector species of veterinary interest: phylogeography of Culicoides imicola (Diptera: Ceratopogonidae) in the Indian Ocean revealing low genetic structure
Source: Parasite. 2026 Sep 14;33:50. doi: 10.1051/parasite/2026044 (PMC13574315; doi:10.1051/parasite/2026044)
Supplement: Supplementary file 2 [file parasite-33-50-s3.pdf]

Table S3: P-distance (below diagonal) and Pairwise FSTs (above the diagonal) between populations using the *COI* and *COII* concatenated

	RE_D	RE_K	RE_I	RE_A	RE_J	MU_K	MU_R	MU_A	MU_C	MU_E	MG_M	MG_S	MG_U	ZA_D	ZA_A
**RE_D**		-0.0081	0.0057	0.0162	0.0388	**0.6567**	**0.6095**	**0.6223**	**0.2796**	**0.5557**	**0.4909**	**0.4738**	**0.5628**	**0.5866**	**0.6338**
**RE_K**	0.00017		0.0320	0.0398	0.0578	**0.6939**	**0.6848**	**0.6612**	**0.3273**	**0.5980**	**0.5617**	**0.5396**	**0.6436**	**0.6319**	**0.6858**
**RE_I**	0.00086	0.00090		-0.0191	0.0131	**0.5168**	**0.5159**	**0.5251**	**0.2197**	**0.4627**	**0.3259**	**0.3298**	**0.3735**	**0.4459**	**0.4654**
**RE_A**	0.00080	0.00084	0.00142		0.0102	**0.5286**	**0.5466**	**0.5406**	**0.2355**	**0.4736**	**0.3383**	**0.3431**	**0.3867**	**0.4692**	**0.4921**
**RE_J**	0.00086	0.00089	0.00150	0.00143		**0.4499**	**0.5207**	**0.4706**	**0.1859**	**0.3993**	**0.3368**	**0.3459**	**0.4041**	**0.4666**	**0.4874**
**MU_K**	0.00271	0.00268	0.00309	0.00300	0.00265		**0.3795**	-0.0006	**0.0971**	-0.0270	**0.4782**	**0.4590**	**0.5208**	**0.4894**	**0.5196**
**MU_R**	0.00496	0.00494	0.00494	0.00492	0.00490	0.00406		**0.3068**	**0.1191**	**0.3145**	**0.2949**	**0.2716**	**0.2541**	**0.2169**	**0.2225**
**MU_A**	0.00324	0.00321	0.00355	0.00349	0.00313	0.00167	0.00394		**0.1038**	0.0110	**0.4679**	**0.4510**	**0.5061**	**0.4529**	**0.4785**
**MU_C**	0.00291	0.00291	0.00324	0.00318	0.00307	0.00282	0.00425	0.00303		**0.0658**	**0.1734**	**0.1662**	**0.1914**	**0.1952**	**0.2043**
**MU_E**	0.00292	0.00290	0.00327	0.00317	0.00287	0.00170	0.00410	0.00196	0.00298		**0.4014**	**0.3866**	**0.4354**	**0.4183**	**0.4389**
**MG_M**	0.00246	0.00249	0.00273	0.00264	0.00272	0.00351	0.00435	0.00383	0.00360	0.00355		-0.0515	-0.0101	**0.1180**	**0.1124**
**MG_S**	0.00258	0.00262	0.00282	0.00273	0.00283	0.00350	0.00422	0.00380	0.00358	0.00353	0.00220		-0.0203	0.0701	0.0656
**MG_U**	0.00301	0.00305	0.00320	0.00308	0.00328	0.00412	0.00461	0.00447	0.00404	0.00409	0.00252	0.00253		**0.1584**	**0.1430**
**ZA_D**	0.00290	0.00292	0.00303	0.00300	0.00308	0.00328	0.00352	0.00340	0.00337	0.00332	0.00232	0.00225	0.00270		-0.0263
**ZA_A**	0.00296	0.00298	0.00309	0.00307	0.00315	0.00342	0.00362	0.00356	0.00347	0.00345	0.00231	0.00224	0.00267	0.00180	

p-distance

FST

dataset (SWIO dataset) of *C. imicola* in the South West Indian Ocean. Significant values are highlighted in bold.
